# Comparative Proteomics Reveals Important Viral-Host Interactions in HCV-Infected Human Liver Cells

**DOI:** 10.1371/journal.pone.0147991

**Published:** 2016-01-25

**Authors:** Shufeng Liu, Ting Zhao, BenBen Song, Jianhua Zhou, Tony T. Wang

**Affiliations:** 1 Center for Immunology and Infectious Diseases, Bioscience Division, SRI International, Harrisonburg, Virginia, 22802, United States of America; 2 College of Pharmacy, University of Michigan, Ann Arbor, Michigan, 48109, United States of America; 3 SLS Global Technical Support, Pall Corporation, Port Washington, New York, 11050, United States of America; 4 Department of Urology, School of Medicine, University of Pittsburgh Medical Center, Pittsburgh, Pennsylvania, 15232, United States of America; University of Washington, UNITED STATES

## Abstract

Hepatitis C virus (HCV) poses a global threat to public health. HCV envelop protein E2 is the major component on the virus envelope, which plays an important role in virus entry and morphogenesis. Here, for the first time, we affinity purified E2 complex formed in HCV-infected human hepatoma cells and conducted comparative mass spectrometric analyses. 85 cellular proteins and three viral proteins were successfully identified in three independent trials, among which alphafetoprotein (AFP), UDP-glucose: glycoprotein glucosyltransferase 1 (UGT1) and HCV NS4B were further validated as novel E2 binding partners. Subsequent functional characterization demonstrated that gene silencing of UGT1 in human hepatoma cell line Huh7.5.1 markedly decreased the production of infectious HCV, indicating a regulatory role of UGT1 in viral lifecycle. Domain mapping experiments showed that HCV E2-NS4B interaction requires the transmembrane domains of the two proteins. Altogether, our proteomics study has uncovered key viral and cellular factors that interact with E2 and provided new insights into our understanding of HCV infection.

## Introduction

HCV is an important human pathogen that primarily infects human hepatocytes and causes chronic liver diseases [1]. This deadly RNA virus encodes ten viral proteins to complete its life cycle. In order to establish a productive infection, HCV structural proteins (core, E1, and E2) and nonstructural proteins (NS2, NS3, NS4A, NS4B, NS5A, NS5B) form complex interaction networks (interactomes) with a myriad of host cellular factors. Viral glycoproteins E1 and E2 together form spikes on the viral envelope, which then engage with cell surface molecules[2–7], including CD81[6], scavenger receptor BI (SR-BI) [7], claudin-1 (CLDN1) [8], occludin (OCLN) [9, 10], epidermal growth factor receptor (EGFR)[8], and cholesterol-uptake receptor Niemann-Pick C1-like 1 (NPC1L1) [9], and trigger the endocytosis of the viral particle[10, 11]. The ectodomain of E2 is the primary ligand that binds aforementioned receptors, whereas transmembrane domain (TMD) of E2 functions in the membrane anchoring, heterodimerization with E1, and ER retention [12–16]. A hydrophobic sequence locating in the TMD of E2 is important for E2 translocation to ER lumen where the glycosylation occurs [14]. Besides mediating viral entry, E2 also interacts with HCV nonstructural protein 2 (NS2) and plays an important role in virus morphogenesis [17]. However, much of E2 biogenesis as well as its role in viral morphogenesis have yet to be understood.

To fill this knowledge gap, we developed a strategy to purify intact E2 complex formed in HCV infected human hepatoma cells and reproducibly identified 85 HCV E2 binding proteins. Our comparative proteomics and functional analyses revealed an important interaction between HCV E2 and the endoplasmic reticulum (ER) protein UGT1, which regulates the production of infectious HCV. Interestingly, another viral protein, NS4B, was also found to interact with E2. Multiple domains of HCV NS4B coprecipitated with HCV E2 and this interaction was abolished when E2 transmembrane domain was removed. Characterizing these interactions in detail would provide a deeper understanding of HCV infection and also potentially present targets for antivirals to disrupt virus biology.

## Materials and Methods

### Cells, reagents, and constructs

The human kidney epithelial cell line Lenti-X 293T was purchased from Clontech. The human liver cell line Huh7.5.1 was provided by Dr. Francis Chisari (Scripps Research Institute) [18]. All cell lines were maintained in DMEM supplemented with 5% penicillin and streptomycin, 1% NEAA, and 10% fetal bovine serum (FBS) (Gemini Bio-Products). Anti-Flag M2 antibody and Rabbit anti-UGGT1 antibody (HPA015127) were purchased from Sigma. Secondary antibodies are purchased from Jackson ImmunoResearch Laboratories.

JFH1-Flag-E2, which expresses Flag-tagged JFH1 E2, has been described previously[19]. pLVX-Flag-UGT1 was generated by replacing the EcoRI-XhoI fragment of pLVX-DFT with PCR fragments generated using forward primer UGT1-FP (5′-gcgaattctgggctgcaagggagacgcgag-3′) and reverse primers UGT1-RP (5′-atatagctcgagtcatttcttacccttgatga-3′). Individual HCV protein was PCR amplified from the JFH1 clone and subcloned in frame after a Flag tag vector (pMIR-DFT).

### Affinity purification of HCV E2 complex

Huh-7.5.1 cells (2 x 10^8^) were infected by JFH1-AM2 and JFH1-Flag-E2-AM2 viruses. On day 3 postinfection, cells were trypsinized and washed with ice-cold phosphate-buffered saline and then Dounce homogenized in 10 ml of immunoprecipitation (IP) buffer (20 mM HEPES [pH 7.5], 150 mM NaCl, 1 mM dithiothreitol, 1 mM EDTA, 0.5% NP-40, 5 mM β-glycerophosphate) supplemented with a protease inhibitor cocktail. Centrifugation-cleared lysates were then subjected to IP with 50 μl anti-Flag M2 affinity resin and rotated 4 hours at 4°C. After four washes with the IP buffer, bound proteins were eluted with Flag peptide (100μg/ml, Sigma) in 100 μl Tris-buffered saline and resolved on SDS/PAGE, and the proteins were visualized by SYRO Ruby staining. Roughly 20 gel slices were excised from either JFH1-AM2 or JFH1-Flag-E2-AM2 lane and subjected for LC-MS/MS.

### LC-MS/MS and data analysis

Gel bands of interest were subjected to in-gel digestion according to established protocols [20]. Briefly, gel bands were destained in 50% acetonitrile in 50 mM NH_4_HCO_3_, pH 8.4 and vacuum dried. Trypsin (20 μg/mL in 25 mM NH_4_HCO_3_, pH 8.4) was added and samples were allowed to incubate on ice for 45 minutes. The supernatant was removed and the gel bands were covered with 25 mM NH_4_HCO_3_, pH 8.4, and incubated at 37°C overnight. Tryptic peptides were extracted from the gel pieces with 70% acetonitrile, 5% formic acid, lyophilized to dryness and resuspended in 10 μL of 0.1% formic acid prior to MS analysis.

Nanoflow reversed-phase liquid chromatography (RPLC) was performed using a Dionex Ultimate 3000 LC system (Dionex Corporation, Sunnyvale, CA) coupled online to an LTQ-Orbitrap XL mass spectrometer (ThermoFisher Scientific, San Jose, CA). Separations were performed using 75 μm i.d. x 360 o.d. x 20 cm long fused silica capillary columns (Polymicro Technologies, Phoenix, AZ) that were slurry packed in house with 5 μm, 300 Å pore size C-18 silica-bonded stationary phase (Jupiter, Phenomenex, Torrance, CA). Following sample injection onto a C-18 trap column (Dionex), the column was washed for 3 min with mobile phase A (2% acetonitrile, 0.1% formic acid in water) at a flow rate of 0.3 μL/min. Peptides were eluted using a linear gradient of 0.34% mobile phase B (0.1% formic acid in acetonitrile) / min for 117 minutes, then to 95% B in an additional 10 min, all at a constant flow rate of 0.2 μL/min. Column washing was performed at 95% B for 20 minutes, after which the column was re-equilibrated in mobile phase A prior to subsequent injections.

The LIT-MS was operated in a data dependent MS/MS mode in which each full MS scan was followed by seven MS/MS scans where the seven most abundant peptide molecular ions are selected for collision-induced dissociation (CID), using a normalized collision energy of 35%. Data were collected over a broad mass to charge (m/z) precursor ion selection scan range of 300–1800, utilizing dynamic exclusion to minimize redundant selection of peptides previously selected for CID. Tandem mass spectra were searched against a combined UniProt human protein database (03/2011) from the European Bioinformatics Institute (http://www.ebi.ac.uk/integr8) and the Hepatitis C virus genotype 2a (isolate JFH-1) protein sequence (UniProt Accession Q99IB8) using SEQUEST (ThermoFisher Scientific). For a fully tryptic peptide to be considered legitimately identified, it had to achieve stringent charge state and proteolytic cleavage-dependent cross correlation (Xcorr) scores of 1.9 for [M+H]1+, 2.2 for [M+2H]2+ and 3.5 for [M+3H]3+, and a minimum delta correlation (ΔCn) of 0.08. The false discovery rate cutoff was set as < 1%. Additionally, peptides were searched for methionine oxidation and cysteine carboxyamidomethylation with a mass addition of 15.99492 and 57.02416, respectively. The obtained proteins were further filtered by removing proteins identified by less than two unique peptides. Finally, those proteins identified from the JFH1-AM2 lane were subtracted from the total proteins that were identified in the JFH1-Flag-E2-AM2 lane (comparative or subtractive approach) in order to obtain the real E2 interacting partners. The purification, LC-MS/MS and subtractive analyses were done three times independently.

Pathway and network analysis on changed heart tissue proteins was performed using Ingenuity Pathway Analysis software (Redwood City, CA).

### shRNA knockdown

Four shRNA clones targeting human UGT1 and the pLKO.1 control plasmid were purchased through TRC consortium from Sigma. To generate lentivirus, 3 μg of shRNA clone, 3 μg of pCMV8.2ΔR, and 1.5 μg VSV-G expression plasmid were transfected into 293T cells in 6-cm plates by lipofectamine 2000 (Invitrogen). Viruses were collected at 48 hours post-transfection and cleared through filtration. 500 μl viruses were added to Huh7.5.1 cells in a 12-well plate and selected by puromycin (0.6 μg/ml). To verify the knockdown efficiency, cell lysates were prepared and analyzed by Western blotting.

### Western Blotting

Briefly, 30 μg of proteins were run on 4–20% precast polyacrylamide gel (Bio-Rad, Hercules, CA) and transferred to nitrocellulose membranes. Membranes were blocked with Odyssey Blocking Buffer (LI-COR, Lincoln, NE) followed by incubation with primary antibodies at a 1:1000 dilutions. Membranes were washed three times with 1X TBS, incubated with IRDye secondary antibodies (LI-COR, Lincoln, NE) for 1 h and washed again to remove unbound antibody. Western blotting images were taken using the ODYSSEY CLx (LI-COR, Lincoln, NE).

### Statistical analysis

Bar graphs were plotted to show mean ± standard deviation (SD) of at least two independent experiments. Statistical analyses were performed using Graphpad Prism 5. A p value of <0.05 in the Student's test was considered statistically significant.

## Results

### Reproducible Identification of HCV E2 Interacting Proteins

The urgent need of prophylactic vaccines and alternative therapies demands for better understanding of the virus life cycle [21–23]. However, previously efforts in depicting the global HCV-Host protein-protein interactome have relied on the yeast two-hybrid assay [24], in which protein-protein interactions form beyond the context of infection. With recent development of cell culture systems supporting the entire HCV life cycle (HCVcc), which can be propagated in human liver cell line Huh7 and its derivatives [2, 18, 25], we are now poised to depict the virus-host interaction networks under physiologically relevant condition.

**Fig 1:**
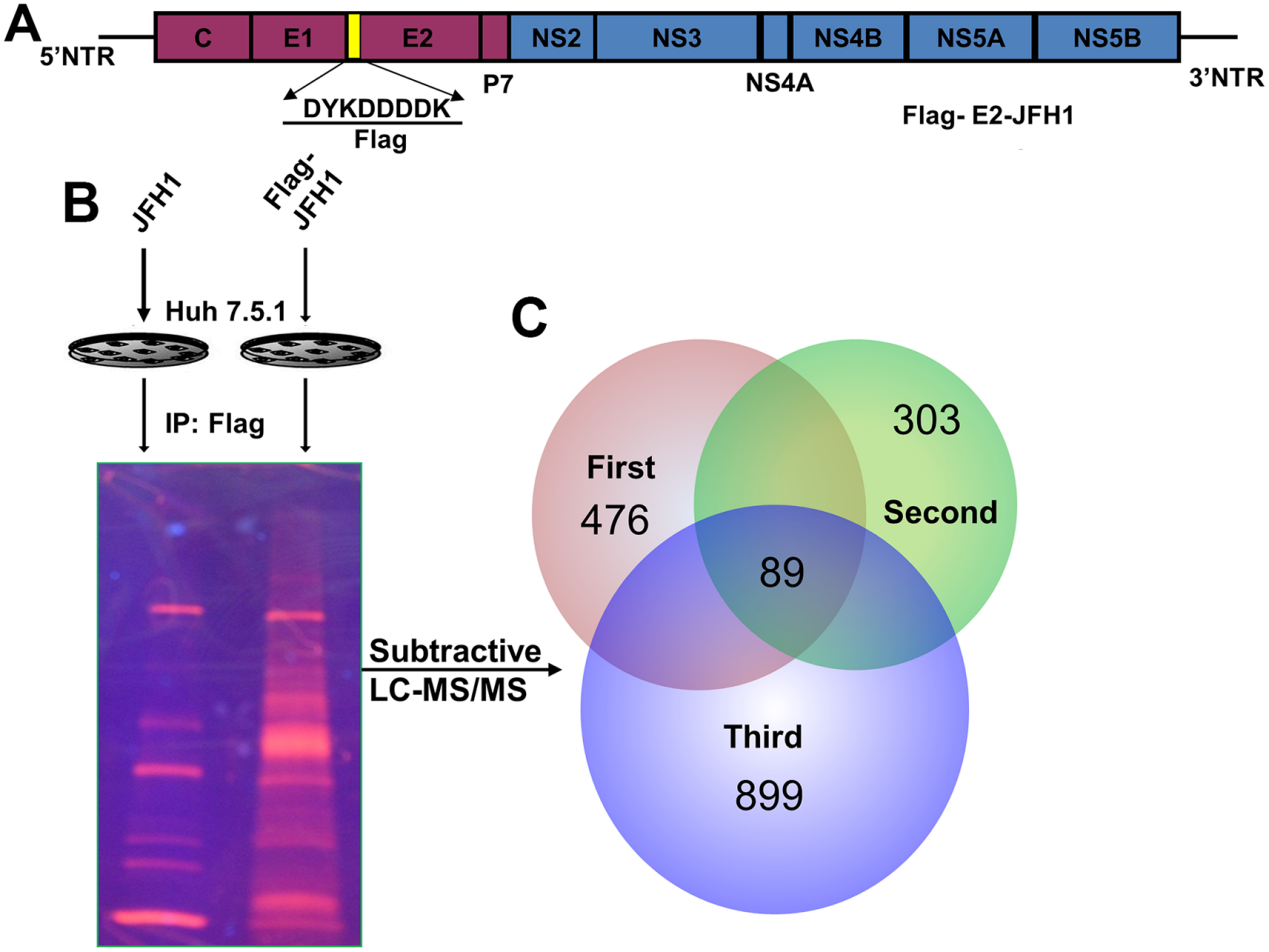
Identification of cellular proteins from HCV E2 complex. Details can be found in Experimental Procedures. (A), Genomic organization of the Flag-E2-JFH1 virus. (B), Schematic of the purification strategy. The infection efficiency was nearly 100% in all three replicates. (C), Venn diagram of 89 proteins (including 4 viral proteins) that were identified in all three trials.

**Fig 2:**
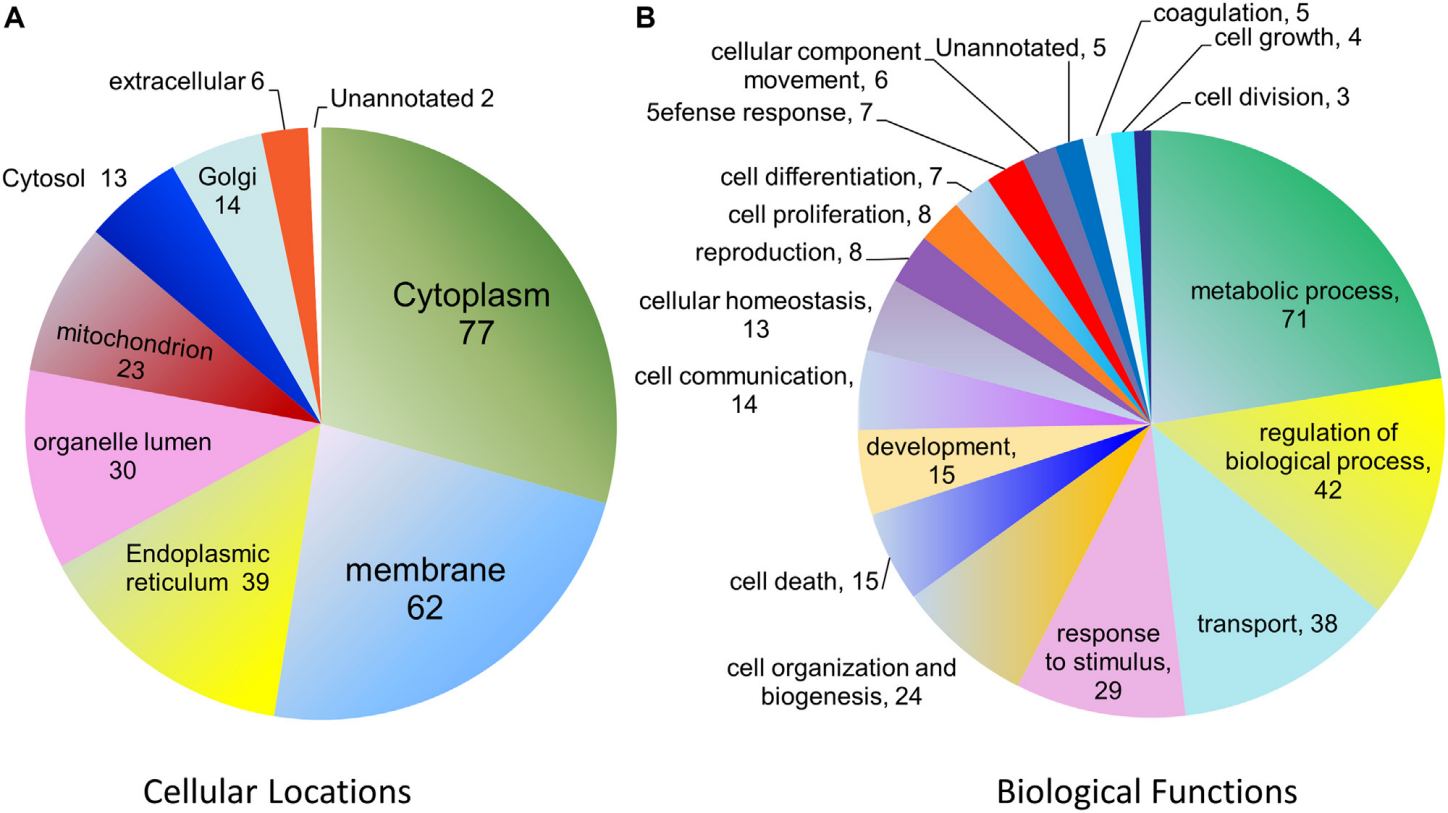
Pie charts showing the frequency of functional groups (analyzed by ProteinCenter, Thermo Scientific) from 85 cellular proteins. Shown in (A) are proteins categorized according to cellular locations. Shown in (B) are proteins categorized according to biological functions. Notably some proteins are classified into more than one location or biological function.

We have previously engineered a Japanese fulminant hepatitis 1 (JFH-1) clone that expresses a FLAG tag in fusion with its glycoprotein E2 (Fig 1A). This clone efficiently infects human Huh7.5 cells and produces infectious virions [19]. Using cell lysates made from the infected Huh7.5 cells, we conducted affinity pull down assays using anti-FLAG agarose resin and identified proteins that associate with E2 (Fig 1B and 1C). The experiments and mass spectrometric analyses were done three times under identical conditions. For database search and protein identification, the set criteria included that a protein must be identified in all three immunoaffinity pull downs with at least two unique peptide matches. In addition, those proteins identified from the control IP samples were subtracted out. Ultimately only those proteins that were identified in all three trials (listed in S1 Table) were subsequently categorized according to cellular distributions and biological functions (Fig 2 and S2 and S3 Tables). The majority of the identified proteins are localized to cytoplasm or membrane. Of note, viral proteins E1, NS2, and NS4B were also pulled down in all three trials.

### Network analysis of HCV E2 associated factors

**Fig 3:**
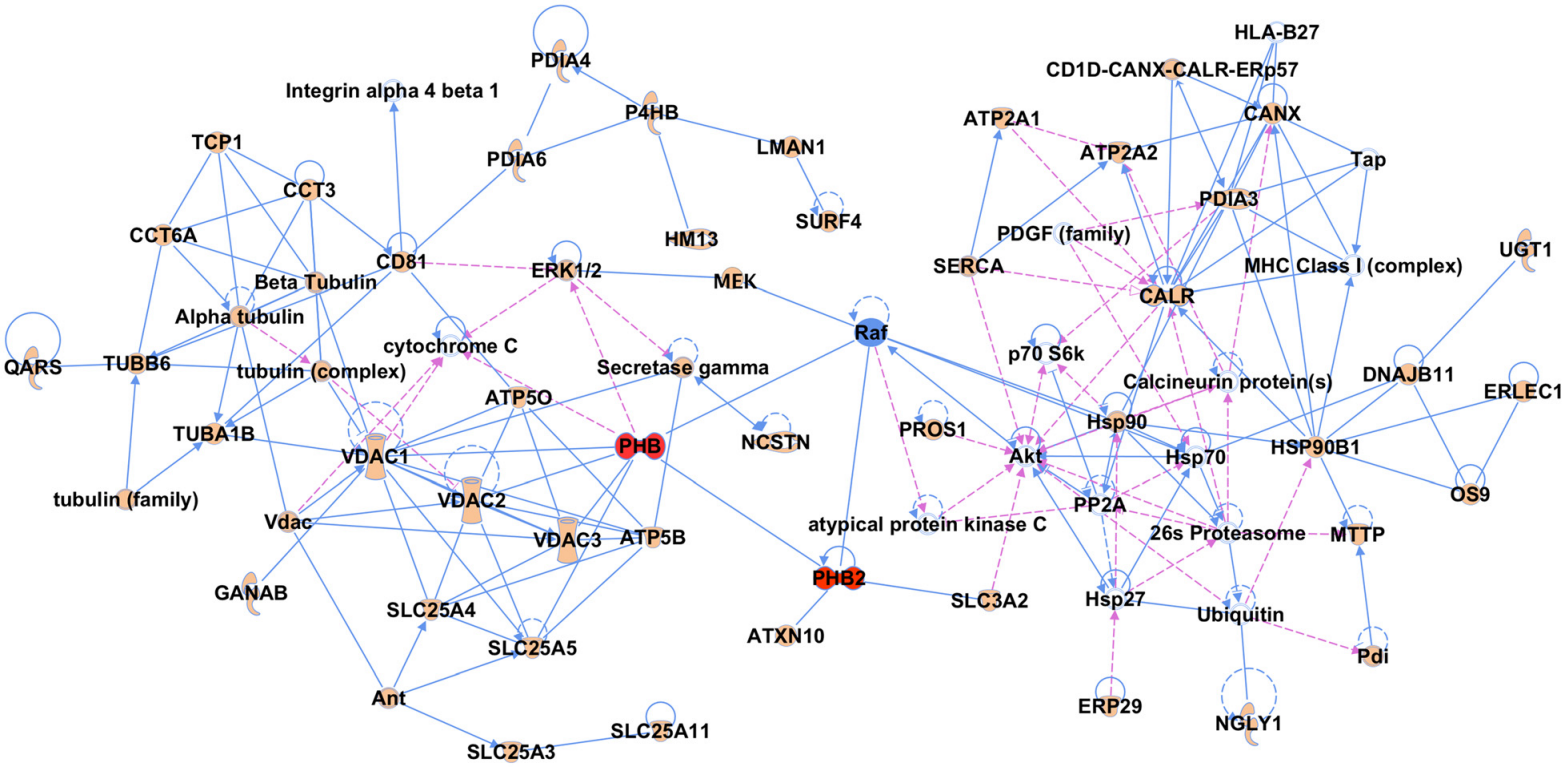
Protein network analysis (using Ingenuity Analysis Software) for PHB1/2 network. PHB1 (PHB) and PHB2 are highlighted in red; proteins labeled in solid shapes were identified in this study except Raf and MEK, which were added according to published data; proteins shown in empty circles were from Ingenuity database. The solid line represents direct interaction; the dashed line represents indirect interaction.

To depict the networks to which the 85 factors are clustered, we used Ingenuity Pathway Analysis (IPA) software to curate information on protein-protein interactions (PPIs) and molecular pathways. Indeed, many of the identified proteins interact with each other according to the database and can be linked to the same protein network, which further validates the success of our affinity purification. Well-represented networks include molecular transport and cell signaling; carbohydrate metabolism, lipid metabolism; cell-to-cell signaling and interaction (S1 Fig). PHB1 and 2 caught our attention due to their abundance in E2 complex and their known roles in regulating Ras-CRaf-MEK-ERK pathway [26] (Fig 3). Subsequent characterization showed that PHB1/2 are essential HCV entry factors and candidate drug targets [27].

### Initial Validation

**Fig 4:**
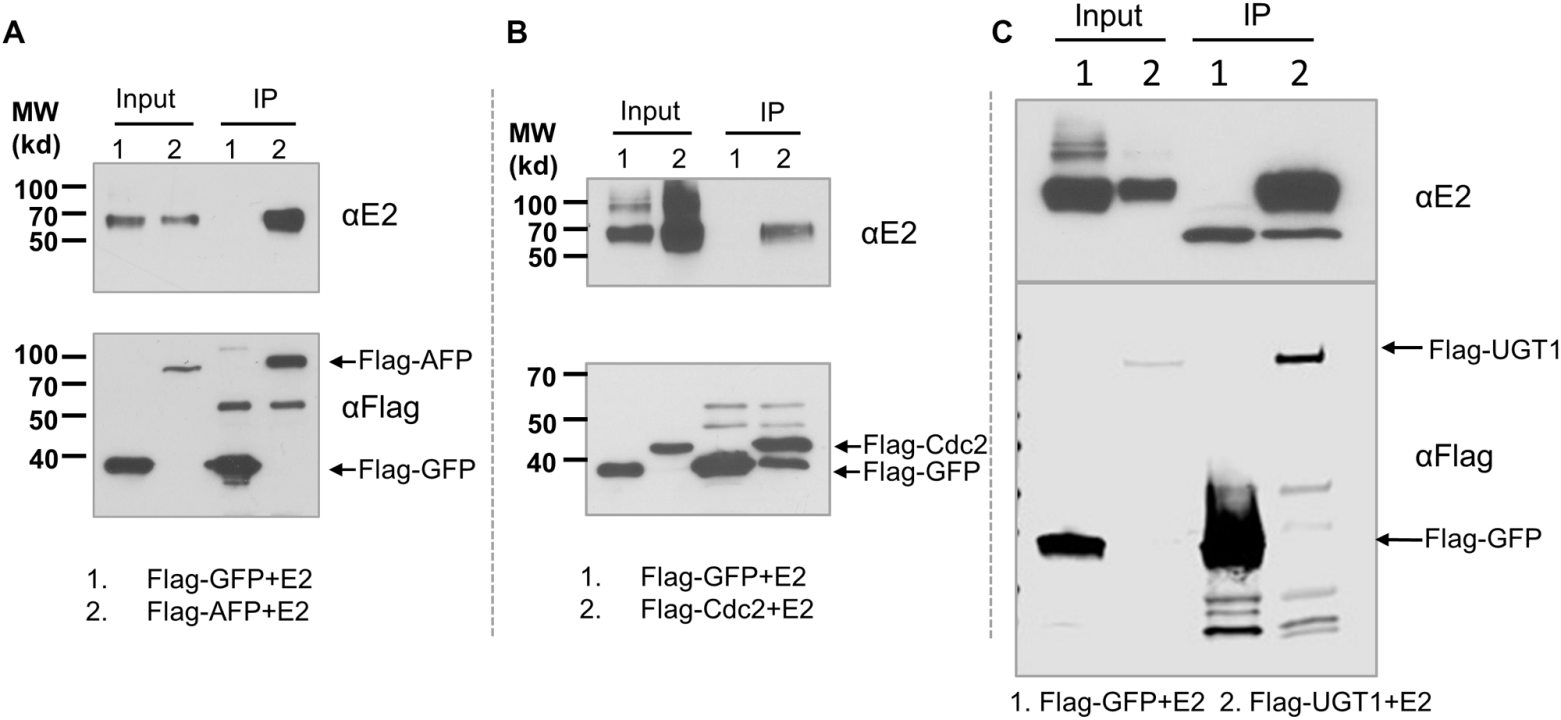
Co-immunoprecipitations of AFP (A), Cdc2 (B), and UGT1 (C) with HCV E2. 293T cells were transfected with indicated plasmids. Whole cell lysates were prepared for immunoprecipitation followed by western blotting. Representative data of three independent experiments are shown.

Among the 85 cellular proteins, endoplasmic reticulum chaperones such as calnexin [28] and calreticulin [29] have been known to interact with HCV glycoproteins and affect their folding; CD81 is a known HCV co-receptor; Cdc2 was previously reported to affect HCV entry[8]. The identification of alpha-fetoprotein (AFP) is interesting because for decades it has been the most widely used biochemical blood test for liver cancer [30]. To further validate the mass spectrometry results and potential interactions between E2 and cellular proteins, we performed reverse immunoprecipitations against selected cellular targets. To this end, we subcloned AFP, UDP-glucose:glycoprotein glucosyltransferase 1 (UGT1), and Cdc2 in a Flag-tagged expression plasmid and co-transfected with a HCV E2 expression plasmid into 293T cells. Whole cell lysates (input) and eluates (IP) from anti-Flag affinity resin were separated by 1D SDS-PAGE. The presence of E2 in three immunoprecipitates was confirmed by Western Blotting using a specific antibody against HCV E2 (Fig 4).

### UGT1 is required for HCV Production

**Fig 5:**
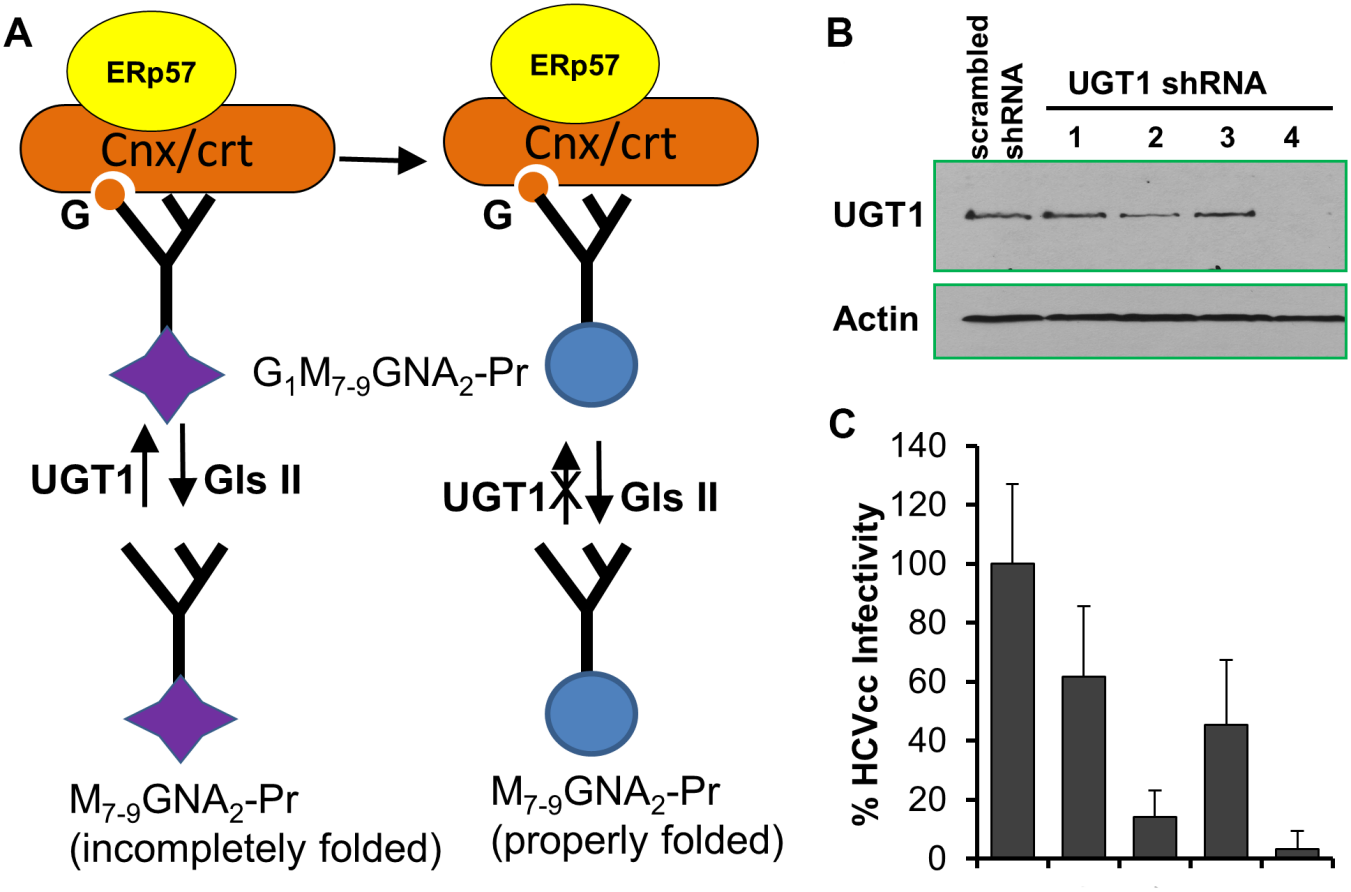
UGT1 knockdown inhibited the production of infectious HCV particles. (**A**) The UGT1-dependent glycoprotein repair and refolding. Incompletely folded proteins carrying N-glycans (M7-9GNA2-Pr) are sensed and re-glucosylated by UGT1, which results in binding to the ER lectin chaperones, calnexin and calreticulin. This prevents the proteins from degradation and allows longer ER retention for further folding. Properly folded proteins are released from Cal/Crt by de-glucosylation by glucosidase II. Abbreviations: GNA,N-acetylglucosamine; M, mannose; G, glucose; Pr, protein; Gls II, glucosidase II;G,glucose;Cnx/crt, calnexin/calreticulin. (B) Western blotting results of UGT1 knockdown in Huh7.5.1 cells. 4 different shRNA lentiviral clones (Purchased from Sigma, clones TRCN0000004520-23) were used to silence UGT1, with clone #4 showing the best silencing effect. (C) Cells from (B) were infected with HCVcc-Luc (MOI 1) and supernatant viruses were collected 48 hrs post infection and tiered for infectivity. Data shown are representatives of three independent experiments.

HCV E2 is synthesized at rough endoplasmic reticulum (ER) and then glycosylated at eleven sites before secretion. It is known that the eleven N-glycans of HCV E2 greatly influence its function in E2 ER-localization, dimerization, maturation, binding to CD81, protein folding, virus entry, assembly, and protection against neutralization, etc [31–34]. One of the E2 interacting partner that was identified in this study, UGT1, is the enzyme that adds a glucose residue from UDP-glucose to an N-linked Man(9)GlcNAc(2)oligosaccharide and thus plays a critical role in repairing folding defects of glycoproteins (Fig 5A).

To investigate the functional role of UGT1 in HCV life cycle, we tested four short-hairpin interfering RNA (shRNA) clones that target human UGT1. Western blotting showed that #2 and #4 shRNA markedly reduced the endogenous level of UGT1 in Huh7.5.1 cells (Fig 5B). Concomitantly, UGT1 knockdown Huh 7.5.1 cells produced significantly less infectious virus (Fig 5C). Presently the exact mechanism for UGT1 to regulate HCV production remains to be determined. However, UGT1 knockdown did not affect HCV entry (data not shown), suggesting that UGT1 is modulating a later step of the viral lifecycle. As an extremely sensitive sensor of the tertiary structure of glycoproteins, UGT1 catalyzes the addition of monoglucose to the defective glycoprotein for repair through an unconventional pathway. Of note, minor defects often occur during glycosylation and can significantly affect the protein folding, maturation and function. Improperly folded glycoproteins will be degraded by proteasomes [35]. Therefore we suspect that UGT1 is required for the proper folding of glycosylated E2, and hence affects its biological function. In fact, a recent study based on HCVcc further demonstrated that several glycans potentially influence HCVcc assembly and infectivity [34]. Given that UGT1is the central enzyme that modifies N-linked glycans for proper folding of glycoproteins, the decreased virus infectivity in UGT1 knockdown cells can be the result of decreased assembly, secretion, decreased specific infectivity of virions as a result of altered E2 glycosylation. Ongoing investigations are dissecting these possibilities.

### HCV NS4B Interacts with E2

**Fig 6:**
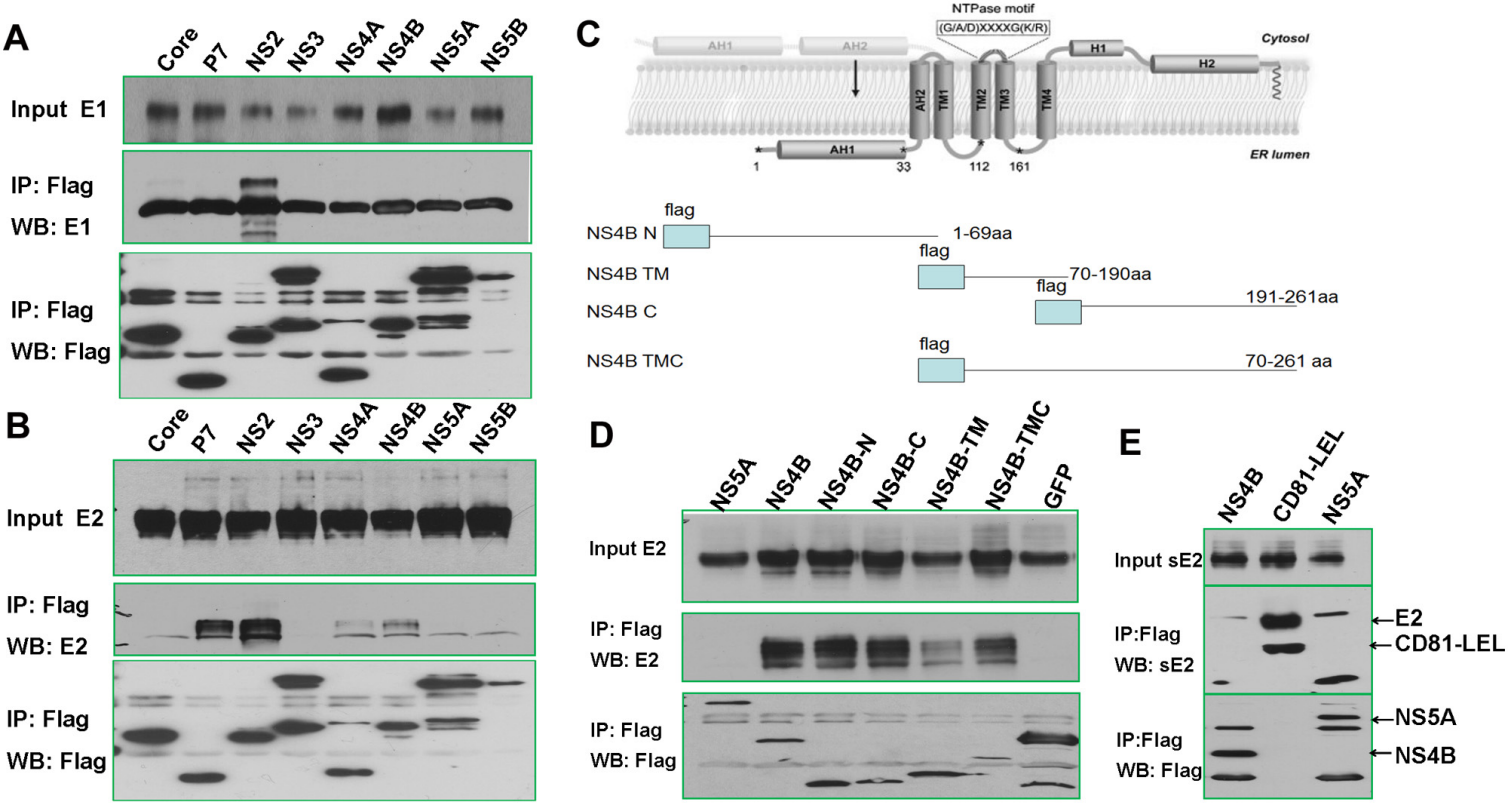
HCV E2 interacts with NS4B. A) 293T cells were co-transfected with indicated flag-tagged HCV protein DNA constructs and a HCV E1 construct. Input cell lysates and immunoprecipitates were blotted with antibodies against HCV E1and flag. Only NS2 robustly interacted with HCV E1. B) Lysates of 293T cells transfected with indicated flag-tagged HCV constructs together with an E2 expressing plasmid were immunoprecipitated with anti-Flag affinity resin. It was observed that p7, NS2, NS4A, and NS4B were able to individually precipitate E2. C) Mapping the domains of NS4B that interact with E2. Topology of HCV NS4B and mutants (modified from ref [36]). D) All NS4B deletion mutants except NS4B-TM robustly precipitated with E2. E) NS4B failed to precipitate soluble E2 (sE2). 293T cells were co-transfected with indicated plasmids expressing flag-NS4B or flag-NS5A or CD81 LEL fused with human Fc (hFc) and soluble E2. CD81-LEL efficiently pulled down sE2, whereas NS4B and NS5A failed to do so. The sE2 (aa 384–661 from H77 clone) lacks the entire TMD and is commonly used in binding studies as E2 substitute [37]. The CD81-LEL contains the large extracellular loop that interacts with sE2 in direct binding assays [38, 39].

The main function of E2 is to bind cellular cell surface molecules, namely the tetraspannin CD81 and scavenger receptor SR-BI [2–7], and trigger the endocytosis of the viral particle [10, 11]. In addition, E2 is believed to play an important role in virus morphogenesis [17]. Interestingly, our affinity pull-down and mass spectrometric analyses identified HCV E1, NS2, and NS4B in E2 complex (S1 Table). To confirm this finding, we expressed individual viral protein with HCV E2 in 293T cells and performed co-IP experiments. It is clear that all of identified proteins co-precipitated with E2 or E1. By contrast, individually expressed NS3, 5A, 5B failed to precipitate with E2 (Fig 6).

Emerging evidence has indicated that p7, NS2, and NS3-NS4A participate in virus particle assembly. In particular, NS2 has been shown to interact with both E1 and E2 during viral assembly and plays an important role in the assembly process [40, 41]. The association between NS4B and E2, on the other hand, has not been reported. HCV NS4B is a 27 kDa ER membrane-associated protein [42] that is conserved in Flaviviridae family and is able to induce alteration of ER membrane and formation of a ‘membranous web’ structure, which provides a platform for the HCV replication complex [43, 44], [43], [45]. The topology of NS4B includes an N-terminal portion (aa 1–69), a central transmembrane part (aa 70–190) with five predicted transmembrane domains and a C-terminal portion (aa 191–261) (Fig 6C).

To characterize the molecular determinants of E2-NS4B interaction, we created deletion constructs containing the N-, C-, TM, TMC domain of NS4B to express Flag tagged proteins. Domain mapping study revealed that the N-terminus and C-terminus regions of NS4B efficiently precipitated with E2 (Fig 6D). The transmembrane domain of NS4B, by contrast, showed reduced capability in co-precipitating E2. Both domains are known to contain the amphipathic helix (AH) which tethers NS4B to ER-membrane [46]. Furthermore, soluble E2 (sE2) that is deprived of its TMD failed to precipitate NS4B, implying the TMD domain of E2 is required for this interaction (Fig 6E). Recent studies have hinted that assembly of infectious HCV may require transient physical interactions between structural and non-structural proteins [47]. NS4B is known to interact with NS5A and 5B in the replication complex [48–52]. Thus it is possible that NS4B serves as the bridge between structural protein E2 and the viral replication complex. In support, HCV NS3, 5A, 5B do not interact with E2, but NS4B is known to interact with NS3, 5A, and 5B to form the viral replication complex [48–52]. NS4B also contains a nucleotide binding motif that may bind viral RNA [53]. It is therefore not hard to envision that NS4B serves as the platform to bring structural proteins to the replication complex where newly synthesized viral RNA can be found for final assembly.

## Discussion

Identifying which host proteins and complexes come into physical contact with the viral proteins is crucial for a comprehensive understanding of how HCV usurps the host's cellular machinery during the course of infection. To our knowledge, this is the first detailed interactome analysis of HCV E2 in the context of infection, which reduces identifying false interactions that only occur in the artificial yeast two-hybrid assay or when a singly overexpressed viral protein is used as a bait in immunoprecipitation. Three independent trials plus stringent criteria for protein identification yielded highly reproducible data that warrant future mechanistic investigations. In a recent study [54], Ramage and colleagues performed tandem affinity purification using individually overexpressed HCV viral protein as bait and combined with siRNA knockdown to generate a map of 139 high-confidence HCV-host protein-protein interactions. Our method differs significantly from that in the referenced study in that ours was performed in an infection setting and the Flag-E2 tagged HCV molecular clone is fully infectious. It is known N-terminally tagged HCV core protein simply leads to a dead virus [55, 56]; hence using individual viral protein tagged at its N-terminus as bait may not preserve authentic viral-host interactions. We believe this explains why several most characterized HCV E2 binding proteins, including its co-receptor CD81, was reproducibly identified in our study but not in the referenced study.

The 85 cellular factors identified in our experiment may not be binding to E2 directly, because we were using a fully infectious molecule clone. HCV E2 interacts with E1 and NS2 as other have published [57–60], it is therefore possible that some of the identified proteins were indirectly associating with E2 through their interactions with these viral proteins. Nevertheless, we subsequently performed co-IP to confirm the interaction with several proteins with E2 in the absence of other viral proteins. When we performed pathway analyses, the majority of the 85 identified cellular proteins can be clustered in same protein networks, supporting the success of our study.

The immediate application of use of the identified interactions is to develop better understanding of the molecular biology of HCV envelope protein E2. Heterodimers between E2 and E1 viral glycoprotein together make up the virus envelop spikes that mediate viral attachment and entry into host cells, and the assembly of infectious virus particles [47]. The function of E2 is influenced by its eleven N-linked glycans. Our study revealed the presence of UDP-glucose: glycoprotein glucosyltransferase 1 (UGT1 or UGCGL1) and HCV NS4B in the same complex. Initial functional characterization now presents preliminary evidence that gene silencing of UGT1 in human hepatoma cell line Huh7.5.1 markedly decreased HCV infectivity of the supernatant virus. UGT1 is the enzyme that catalyzes the addition of a glucose residue from UDP-glucose to an N-linked Man(9)GlcNAc(2)oligosaccharide and thus plays an important role in repairing minor defects in glycoprotein folding [35]. Based on these data and the literatures, it is possible that UGT1 modulates the production of infectious virus particles by affecting the proper folding of HCV E2. Here we also show that multiple regions of HCV NS4B co-precipitated with HCV E2 and the interaction was abolished when E2 transmembrane domain was removed. Previous studies have reported that various genetic interactions exist between structural and non-structural sequences [47]. The NS4B-E2 interaction may contribute to the assembly of infectious HCV particles. Future characterization will be needed to address these possibilities.

Our E2 interaction map now provides an excellent opportunity to look for druggable targets. In a separate publication, we described a detailed validation and functional characterization of E2-prohibitin interaction [27]. In the study, we demonstrated that not only the two newly identified E2-interacting proteins, prohibitins, are essential to viral entry, but serve as cellular targets for a novel class of small molecules to block HCV infection. Overall, our study revealed important virus-host interactions that regulate HCV E2 biogenesis and function and serve as potential targets for drug intervention.

## Supporting Information

S1 Fig Protein network analysis (using Ingenuity Analysis Software) for proteins listed in S1 Table and enriched in carbohydrate and lipid metabolic pathways (A) and cell-to-cell signaling and interaction pathways (B). (TIF)

S1 Table List of the HCV E2 co-purified proteins identified by Nanoflow RPLC-MS/MS. (XLS)

S2 Table Cellular location of identified HCV E2 co-purified proteins. (XLS)

S3 Table Biological Function of identified HCV E2 co-purified proteins. (XLS)
