# Supplementary figures and images for: Comparative Proteomics Reveals Important Viral-Host Interactions in HCV-Infected Human Liver Cells
Source: PLoS One. 2016 Jan 25;11(1):e0147991. doi: 10.1371/journal.pone.0147991 (PMC4726516; doi:10.1371/journal.pone.0147991)

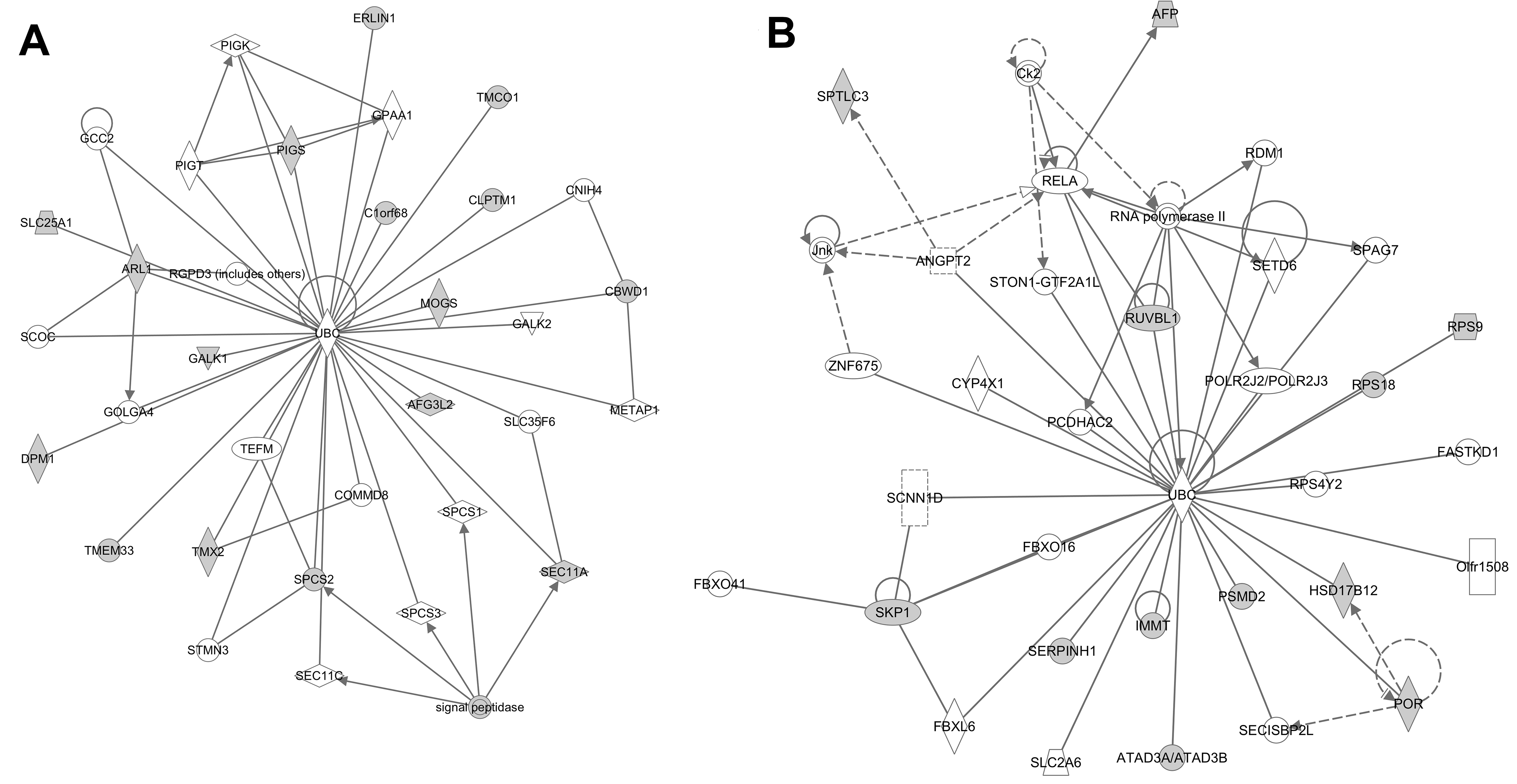

Supplement: S1 Fig — (TIF) [file pone.0147991.s001.tif]
